# A Rare Case of Primary Renal Liposarcoma

**DOI:** 10.7759/cureus.117917

**Published:** 2026-10-14

**Authors:** Anthony Anzano, Jacobi Korus, Vinay Saggar, Benjamin Hong, Koreem A Anderson

**Affiliations:** 1 Department of Emergency Medicine, NewYork-Presbyterian Hospital-Columbia and Cornell, New York, USA; 2 Department of Emergency Medicine, Columbia University College of Physicians and Surgeons, New York, USA

**Keywords:** abdominal mass, ct abdomen and pelvis, leg swelling, primary renal liposarcoma, primary retroperitoneal tumors, surgical resection

## Abstract

Within the broad category of retroperitoneal tumors, primary renal liposarcoma represents an exceedingly rare entity, accounting for less than 1% of tumors. Presentations are often nonspecific, but providers should focus on initial stabilization and consultation with specialists. We present a rare case of primary renal liposarcoma presenting with nonspecific symptoms. A 79-year-old female presented with an abdominal mass, nausea, and bilateral lower extremity edema. Computed tomography (CT) revealed a heterogeneous right retroperitoneal mass measuring 20.6 x 14.9 x 16.0 cm. A subsequent biopsy showed de-differentiated liposarcoma positive for MDM2 (via amplification testing) and CD34 (atypical for this entity). The patient was ultimately discharged and had extensive outpatient resection done. The diagnostic pathway for primary renal liposarcoma begins with a CT, followed by tissue biopsy. Unfortunately, renal liposarcomas carry a poor prognosis because these tumors grow remarkably large due to the ability of the retroperitoneum to accommodate them, and the location itself causes anatomical constraints to surgery. This case highlights that even in the absence of severe clinical complaints, malignant pathology cannot be ruled out. The emergency department (ED) has become an increasingly important pathway for cancer diagnosis, and emergency medicine (EM) physicians play an essential role in initiating multidisciplinary care of patients with aggressive malignancies.

## Introduction

Renal masses encompass a broad spectrum of benign and malignant lesions, the most common of which is renal cell carcinoma (RCC). With the growing use of cross-sectional imaging, their detection has increased over the past decades [1]. Interestingly, a substantial proportion of renal masses are diagnosed incidentally or can present with nonspecific symptoms, such as abdominal pain, flank pain, and vomiting [2]. This trend highlights the importance of risk stratification and leads to a complicated, multifactorial approach for the management of these patients [3-4]. Despite advances in imaging, differentiation between renal neoplasms remains challenging, as entities such as angiomyolipoma, oncocytoma, and RCC subtypes often demonstrate overlapping radiographic features and frequently require tissue diagnosis [5-7]. From the emergency provider's standpoint, initial management focuses on addressing possible complications of these lesions, such as hemorrhage or obstructive uropathy, and emphasizing close follow-up with outpatient urology and oncology [4].

Within the broader category of retroperitoneal tumors, primary renal liposarcoma represents an exceedingly rare entity, accounting for less than 1% of renal tumors with an estimated incidence of 0.53 cases per million people annually [8-9]. Tumors typically arise from perirenal or renal sinus fat and may reach substantial size before detection due to the capacity of the retroperitoneum to accommodate large masses. Presentations are often nonspecific, including abdominal fullness, flank pain, weight loss, or an incidental mass on imaging [10-11]. Few case reports of primary renal liposarcoma are published in the current literature, and in the absence of standardized diagnostic algorithms for them, management is largely based on the guidelines for the larger category of retroperitoneal sarcomas, with complete surgical resection as the mainstay of therapy and a nuanced role for systemic therapy and radiotherapy based on subtype and resectability [12]. 

We present a rare case of primary renal liposarcoma presenting with nonspecific symptoms of an abdominal mass, nausea, and bilateral lower extremity edema. Given the limited number of primary renal liposarcoma cases described in the literature, this report offers a rare glimpse at the presentation, management, and treatment of the disease. The case highlights the importance of recognizing subtle manifestations of retroperitoneal malignancy and serves as a reminder of the critical role emergency medicine (EM) physicians play in expediting diagnosis and multidisciplinary care.

## Case presentation

The patient was a 79-year-old female with a past medical history of hypertension and hyperlipidemia, who initially presented to the emergency department (ED) in February 2026 for evaluation of an abdominal mass, nausea, and bilateral lower extremity edema. She stated that since November 2025, she had been experiencing spells of nausea and progressively worsening lower extremity edema. As time went on, the patient reported slowly beginning to feel a palpable abdominal mass, after which she went to her physicians internationally in January 2026, who recommended going to the United States (US) for further workup. The patient's ED visit was intended to establish care in the US and undergo further evaluation and treatment of the mass. She denied any other concurrent symptoms such as chest pain, shortness of breath, vomiting, diarrhea, or other infectious symptoms.

On initial presentation to the ED, the patient was hemodynamically stable with normal vital signs. On physical exam, a palpable mass in the right lower quadrant was present, with associated tenderness to palpation, as well as pitting edema in the bilateral lower extremities. Labs were notable for an elevated creatinine to 1.47 mg/dL (1.23 mg/dL on her last set of labs) and anemia to 10.8 g/dL. Given that the patient presented to the department with a known malignancy, the decision was made to obtain a CT chest and CT abdomen/pelvis with contrast to evaluate the mass and any associated metastases. CT abdomen and pelvis (CTAP) revealed a large, multilobulated, heterogeneous right retroperitoneal mass in the right hemiabdomen measuring 20.6 x 14.9 x 16.0 cm, with significant mass effect (Figures 1-2).

**Figure 1:**
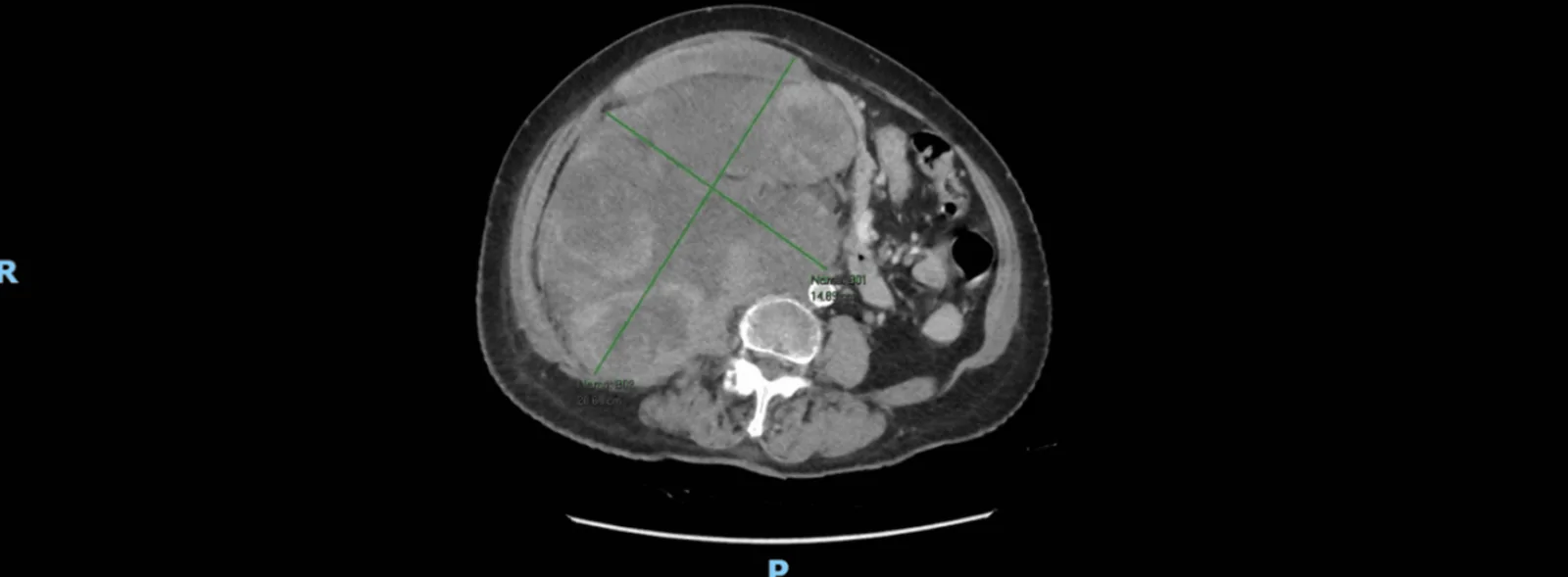
Transverse view Depicted here is a large heterogeneous right retroperitoneal mass measuring 20.6 x 14.9 x 16.0 cm, appearing to involve and arise from the right kidney.

**Figure 2:**
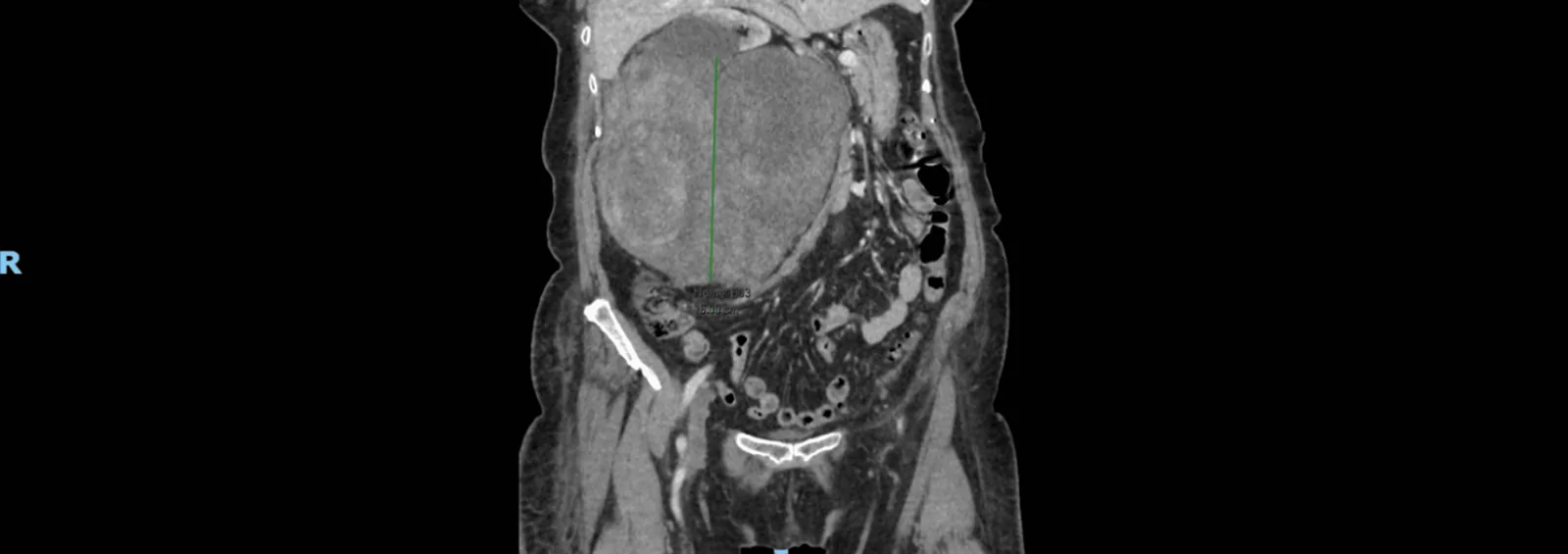
Coronal view Another view depicting the large, heterogeneous right retroperitoneal mass with marked mass effect on surrounding structures and inflammatory fat stranding.

The patient was ultimately admitted to the hospital for an expedited workup.

During admission, solid oncology was consulted and believed the mass was likely a sarcoma. The decision was made to consult Interventional Radiology (IR) for biopsy to obtain pathologic and molecular studies, as well as General Surgery for possible resection. Lower extremity edema was attributed to venous compression rather than new heart failure, as the patient had a normal proBNP and transthoracic echocardiogram (TTE) while inpatient. MRV revealed a complete collapse of the IVC and non-visualization of the right renal vein, concerning for direct invasion versus high-grade compression without concurrent metastasis. MRA revealed displacement of the superior mesenteric and common hepatic arteries without clear invasion. Her biopsy eventually revealed de-differentiated liposarcoma positive for MDM2 (determined by amplification testing) and CD34 (which is atypical for this entity), and negative for S100, DOG-1, and CKAE1/AE3. Ultimately, the patient was stable for discharge.

The patient's admission ultimately led to more rapid surgical planning, and within a few weeks after hospitalization, she underwent mass resection, right nephrectomy, right adrenalectomy, and partial diaphragm resection with primary repair. Although her initial post-operative course was complicated by persistent anemia requiring blood transfusion and close hemodynamic monitoring, the patient has been recovering well since then in subacute rehab and is currently having ongoing discussions with oncology about the role of neoadjuvant therapy. 

## Discussion

While the primary renal liposarcoma described in this case is exceedingly rare, the ED has become an increasingly important pathway for cancer diagnosis broadly. Data suggest that approximately one-third of patients diagnosed with cancer will have used the ED in the 90 days before their diagnosis, with over half of these patients being admitted to the hospital on that ED visit [13-14]. The increasing use of CT imaging in the ED has contributed to the detection of occult malignancies; in fact, a systematic review found that incidental findings can occur in up to 31% of scans performed in the ED, many of which will require malignancy workups [2].

A review by Delamare et al. [15] highlights the three major ways that the ED can contribute to cancer diagnoses: (1) incidental findings of suspected cancer for unrelated complaints, (2) presentation of cancer with acute signs and symptoms requiring emergency care, and (3) acceleration of a cancer diagnosis and further care. In this case, the ED served in the third function - establishing care in a new healthcare system and expediting the workup of an incompletely characterized malignancy. EM physicians play an essential role in initiating the multidisciplinary care these patients will inevitably require, which can include surgeons, oncologists, and palliative specialists.

The diagnostic pathway for retroperitoneal sarcoma typically begins with cross-sectional imaging. Per the National Comprehensive Cancer Network (NCCN) guidelines, CT with contrast is preferred for retroperitoneal tumors, providing critical information on tumor size, heterogeneity, and relationship to surrounding structures [16]. In this case, CT abdomen/pelvis revealed a large, multilobulated, heterogeneous right retroperitoneal mass with significant mass effect, highly suggestive of malignancy and requiring further workup. Following imaging, tissue diagnosis is typically the next step in treatment planning. Image-guided core needle biopsy is the preferred approach for retroperitoneal masses and should avoid the free intra-abdominal space to avoid further tumor seeding [16]. CT-guided biopsy has been shown in some studies to have 77% accuracy for identifying final histology in abdominal sarcomas, with sensitivity of 79% and specificity of 100% for liposarcomas specifically. However, accuracy is notably lower for dedifferentiated liposarcomas (62.5%) when compared to well-differentiated liposarcomas (75%), highlighting how final diagnosis can be difficult with this patient's subtype [17]. 

Retroperitoneal sarcomas carry a worse prognosis than other sarcomas, with five-year survival ranging from 40% to 50% [18]. Several factors contribute to this poor prognosis. First, these tumors remain occult for a long time and can grow to be remarkably large due to this area's ability to accommodate large masses [19]. 50% of these tumors will grow to greater than 20 cm by the time of initial presentation [19]. This patient's tumor measured 20.6 x 14.9 x 16.0 cm, consistent with this pattern of late detection. Second, the retroperitoneal location itself is an independent adverse prognostic factor. There are also anatomical constraints to surgery in this region, with approximately 75% of patients dying of locally recurrent disease without distant metastasis [19]. Third, the result of histologic subtype can affect anticipated clinical course. Dedifferentiated liposarcoma demonstrates significantly worse outcomes, with five-year overall survival around 50% versus 82% for well-differentiated [20]. 

Complete surgical resection is the cornerstone of treatment and the only potential curative option for retroperitoneal sarcoma [12]. The best outcomes are achieved with margin-negative resection, with five-year disease-free survival found to be 50% with margin-negative compared to 28% for incomplete resection [19]. The role of neoadjuvant therapy remains nuanced and subtype-dependent. For unresectable tumors, systemic therapy and/or radiotherapy may be used as an effort to achieve resectability. If this remains unachievable, options such as palliative radiotherapy, palliative surgery, or supportive care should be considered [16].

## Conclusions

This case highlights that even in the absence of severe clinical complaints, malignant pathology cannot be ruled out. The ED has become an increasingly important pathway for cancer diagnosis broadly; in this case, the ED served as a catch net for establishing care for a rare malignancy that carries an exceptionally poor prognosis. Although likely not part of the EM provider’s standard differential, once an aggressive malignancy is identified, EM physicians play an essential role in initiating multidisciplinary care for these patients.
